# Determination of the Pathological Features of NPC1 Variants in a Cellular Complementation Test

**DOI:** 10.3390/ijms20205185

**Published:** 2019-10-19

**Authors:** Xiao Feng, Claudia Cozma, Supansa Pantoom, Christina Hund, Katharina Iwanov, Janine Petters, Christin Völkner, Claudia Bauer, Florian Vogel, Peter Bauer, Frank U. Weiss, Markus M. Lerch, Anne-Marie Knospe, Andreas Hermann, Moritz J. Frech, Jiankai Luo, Arndt Rolfs, Jan Lukas

**Affiliations:** 1 Translational Neurodegeneration Section „Albrecht-Kossel“, Department of Neurology, University Medical Center Rostock, University of Rostock, 18147 Rostock, Germany; xiao.feng@uni-rostock.de (X.F.); supansa.pantoom@med.uni-rostock.de (S.P.); christina.hund@med.uni-rostock.de (C.H.); katharina.iwanov@med.uni-rostock.de (K.I.); janine.petters@med.uni-rostock.de (J.P.); christin.voelkner@med.uni-rostock.de (C.V.); anne.knospe@neuroproof.com (A.-M.K.); andreas.hermann@med.uni-rostock.de (A.H.); moritz.frech@med.uni-rostock.de (M.J.F.); jiankai.luo@med.uni-rostock.de (J.L.); 2 Centogene AG, 18055 Rostock, Germany; claudia.cozma@centogene.com (C.C.); claudia.bauer@centogene.com (C.B.); florian.vogel@centogene.com (F.V.); peter.bauer@centogene.com (P.B.); arndt.rolfs@centogene.com (A.R.); 3 Department of Medicine A, University Medicine Greifswald, 17489 Greifswald, Germany; ulrich.weiss@uni-greifswald.de (F.U.W.); Markus.Lerch@med.uni-greifswald.de (M.M.L.); 4 Center for Transdisciplinary Neurosciences Rostock (CTNR), Rostock University Medical Center, University of Rostock, 18147 Rostock, Germany; 5 German Center for Neurodegenerative Diseases (DZNE), Research side Rostock, 18147 Rostock, Germany

**Keywords:** variant of unknown significance, lipid metabolism, pharmacological chaperone, liver disease

## Abstract

Niemann-Pick Type C (NP-C) is a rare disorder of lipid metabolism caused by mutations within the *NPC1* and *NPC2* genes. NP-C is a neurovisceral disease leading to a heterogeneous, multisystemic spectrum of symptoms in those affected. Until now, there is no investigative tool to demonstrate the significance of single variants within the *NPC* genes. Hence, the aim of the study was to establish a test that allows for an objective assessment of the pathological potential of *NPC1* gene variants. Chinese hamster ovary cells defective in the *NPC1* gene accumulate cholesterol in lysosomal storage organelles. The cells were transfected with NPC1-GFP plasmid vectors carrying distinct sequence variants. Filipin staining was used to test for complementation of the phenotype. The known variant p.Ile1061Thr showed a significantly impaired cholesterol clearance after 12 and 24 h compared to the wild type. Among the investigated variants, p.Ser954Leu and p.Glu1273Lys showed decelerated cholesterol clearance as well. The remaining variants p.Gln60His, p.Val494Met, and p.Ile787Val showed a cholesterol clearance indistinguishable from wild type. Further, p.Ile1061Thr acquired an enhanced clearance ability upon 25-hydroxycholesterol treatment. We conclude that the variants that caused an abnormal clearance phenotype are highly likely to be of clinical relevance. Moreover, we present a system that can be utilized to screen for new drugs.

## 1. Introduction

Niemann-Pick Type C disease (NP-C, OMIM # 257220) is a rare autosomal recessive lipidosis and belongs to the class of lysosomal storage disorders (LSDs). In most cases, the disease is caused by mutations in the gene encoding the cholesterol transporter NPC1 (95%). Mutations in the *NPC2* gene are responsible for the remaining 5% of those affected. In both cases, the clinical manifestations are indistinguishable [1]. NP-C is a neurovisceral disease and leads to a heterogeneous spectrum of symptoms in affected individuals. The visceral manifestations mainly involve the liver, the spleen, and, to a lesser extent, also the lung. Misdiagnoses with other LSDs and hereditary diseases in which the liver is affected can occur frequently [2,3].

The *NPC1* gene (OMIM *607623) comprises 25 exons. There are over 400 *NPC1* mutations known to cause NP-C disease [4]. However, numerous sequence alterations that are considered benign have also been described to date [5]. The pathogenic potential of individual gene variants may be masked due to recessive disease allele carrier status. Moreover, novel gene variants of unknown significance (GVUS) associated with atypical and late-onset NP-C progression may go unnoticed with the consequence of a delayed diagnosis. A deeper molecular investigation of the gene variants found in these atypical courses can be a step towards the systematic classification of the variants.

The cell biological phenotype of NP-C involves cholesterol accumulation in lysosome-like storage organelles (LSOs) [6]. The visualization of the intracellular cholesterol deposits in patient’s skin fibroblasts using the polyene macrolide antibiotic filipin for fluorescence microscopic examination [7] is still the gold standard in laboratory Niemann-Pick type C diagnostics [5]. However, this test is time-consuming, cost-intensive, and, worst of all, fairly insensitive leading to ambiguous results when lysosomal cholesterol accumulation is attenuated [8]. Further diagnostic methods such as biomarkers are being developed, the detection of which does not require an invasive skin biopsy but can be measured in human blood plasma [9,10]. However, these tests can only be performed in diagnostic laboratories with special mass spectrometry technology.

The technology for genome and exome sequencing has become accessible and affordable for more and more laboratories in recent years. This enables clinical researchers to implement genetic testing for NP-C in newborn screenings [11] and “at-risk-populations” like neurodegeneration [12] and ataxia [13] of unexplained etiology leading to a growing number of gene variants identified. In the same way, we examined a cohort of 149 patients with either non-alcoholic fatty liver disease (NAFLD) or liver cirrhosis (LC) of unexplained etiology for genetic causes. To this end, a panel of 330 genes associated with liver disease was defined and analyzed using a whole genome sequencing approach. We identified a number of *NPC1* gene variants, four out of five of which were classified as GVUS. NP-C disease could be ruled out for these patients, because only mono-allelic gene alterations were observed. The variants were investigated for a metabolic phenotype in order to determine carrier status and, eventually, risk potential to promote the liver pathology. We used Chinese hamster ovary (CHO) cells lacking the *NPC1* cholesterol transporter (CHO^NPC1^) due to a partial deletion in exon 4 [14] to create a complementation assay that indicates the potential of *NPC1* variants to release accumulated cholesterol from LSOs. To sum up, we developed a highly reproducible test setup to investigate *NPC1* variants.

## 2. Results

*NPC1* gene variants were identified in a patient cohort with liver pathology. Four of the variants, p.Gln60His (rs145666943), p.Val494Met (rs199812609), p.Ser954Leu (rs543206298), and p.Glu1273Lys (rs969680897) have been identified in NAFLD patients, p.Ile787Val (rs202046984) was found in a patient with liver cirrhosis. All mutation carriers identified were heterozygous carriers of a single *NPC1* variant. These variants were not found in the control cohort of the study and are very rarely found in the overall population as referenced in the CentoMD database and gnomAD (Table 1).

### 2.1. Mapping of the Gene Variants on the Protein Structure

**Figure 1:**
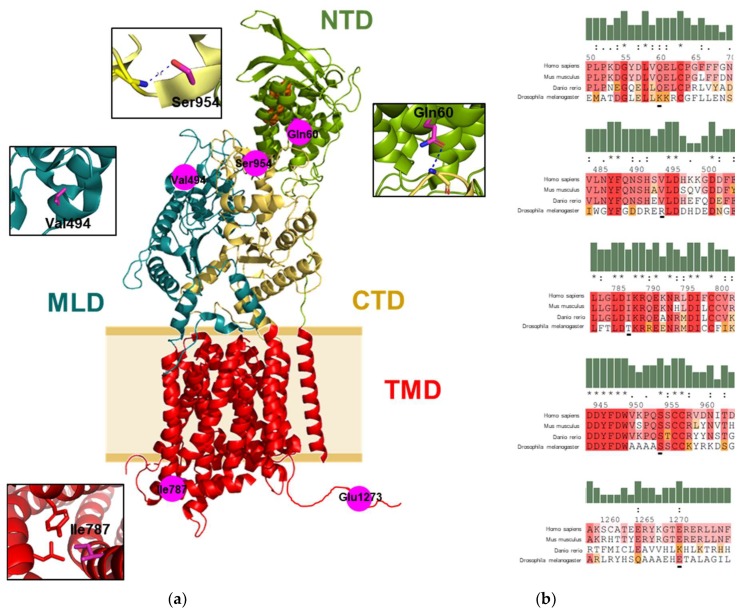
Sequence and structure information on variant NPC1 protein. (**a**) The structure of NPC1 (PDB: 3JD8) indicated the location of the studied residues on NPC1 structure. Functional domains of the protein are indicated in different colors. NTD, N-terminal domain; MLD, middle luminal domain; CTD, C-terminal domain; and TMD, transmembrane domain. The cholesterol molecule is shown as an orange sphere. Intra and interdomain contacts of the mutation residues are shown in the insets. Hydrogen bonds are indicated as a blue dashed line. Protein structural analysis was performed with PyMol software (Schrödinger LLC, Mannheim, Germany). (**b**) Protein sequence alignment of the studied NPC1 residues from human with the NPC1 from mouse (Mus muculus), zebrafish (Danio rerio), and fruit fly (Drosophila melanogaster), the residues are shaded based on their levels of conservation in the alignment. The sequences were aligned using multiple sequence viewer (Schrödinger LLC, Mannheim, Germany).

p.Ser954Leu is classified “pathogenic” applying ACMG variant classification rules [16] and repeatedly reported to be an NP-C related variant [17]. There is insufficient evidence to classify the remainder of the variants other than GVUS. Allele frequency, clinical significance, and a summary of different in silico prediction tools of the found *NPC1* variants were compiled in Table 1. We then aimed to obtain preliminary information about the variants found by protein structure analysis. Interestingly, every amino acid substitution is located on a different functional domain of the NPC1 protein (Figure 1a). Intra and interdomain interaction of the amino acid residues with neighboring residues provides information of potential effects of the variants studied. The Oε1 of Gln60 at the N-terminal domain (NTD) form inter-hydrogen bonding with the N of the main chain of Gly986 at the C-terminal domain (CTD). Substitution of Gln60 to histidine will weaken the hydrogen bond interaction. Val494 is located at the middle luminal domain (MLD) without interaction to the neighboring residues. Therefore, substitution of this residue to methionine has likely no significant structural impact. The Ile787 is located on the cytosolic side of the transmembrane domain (TMD). It forms a hydrophobic patch with Tyr709, Leu783, and Leu720 and its substitution with valine will weaken the hydrophobic interaction. The Oγ of Ser954 is located at the CTD, it forms an intra-hydrogen bond with the amino group of the mainchain of Ser955 within the same domain. Therefore, substitution of Ser954 with leucine will disrupt the hydrogen bond interaction. The Glu1273 located at the C-terminal flexible loop. As there is no structure data available on this loop, there is no further evidence on potential abolishment of functional interactions between residues when lysine substitutes for glutamate here. However, it must be noted that zebrafish orthologue of NPC1 carries a lysine at this position (Figure 1b). Altogether, the respective residues show high inter-species preservation, especially Ser954.

### 2.2. p.Ile1061Thr Has an Impaired Ability to Eliminate Cholesterol from LSOs

**Figure 2:**
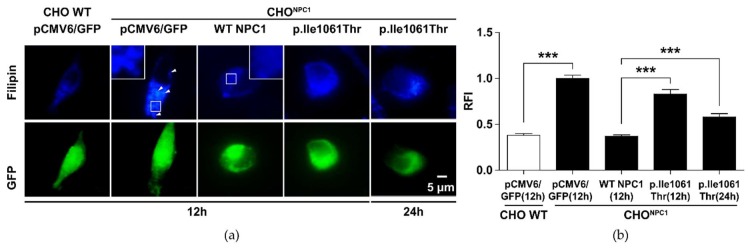
Reduced cholesterol clearance in *NPC1*-deficient Chinese hamster ovary (CHO) cells expressing variant p.Ile1061Thr *NPC1*. CHO^NPC1^ cells develop a phenotype of cholesterol accumulation in lysosome-like storage organelles (LSOs). This phenotype can be corrected by gene transfer of wild type *NPC1* cDNA using liposome-mediated cell transfection. (**a**) Fluorescence microscopic images. The blue staining visualizes cellular filipin-bound cholesterol. The arrowheads indicate LSOs. The green signal shows positively transfected cells that could be used for filipin quantification by either indicating GFP signal (control vector transfection) or NPC1-GFP fusion protein signal. (**b**) Quantification of the positive cholesterol signal. The y-axis shows the relative fluorescence intensity (RFI) signal as arbitrary units. Values are shown as mean ± SD. Asterisks indicate statistical significance: ***p < 0.001.

The aim of the study was to demonstrate the cholesterol transport damage of *NPC1* variants in a complementation assay in *NPC1* deficient CHO cells (CHO^NPC1^). To this end, the frequent *NPC1* variant c.3182T>C (p.Ile1061Thr) was used as an exemplary mutation to establish the assay. This defective NP-C disease-associated variant correlates with the classic juvenile phenotype although genotype-phenotype correlation is not very distinctive [18]. The CHO^NPC1^ cells show an altered filipin signal compared to the paternal wild type CHO line (“WT CHO**”**), which indicates cholesterol accumulation in LSOs (Figure 2). We then transfected the CHO^NPC1^ cells with a WT NPC1-GFP vector construct and quantified the filipin signal in all positively transfected cells. Filipin appeared fully normalized to the level of the paternal cells after 12 h. This also proves that GFP fusion to NPC1 does not influence its capacity to eliminate cholesterol from LSOs. Transfection with p.Ile1061Thr NPC1 caused only partial clearance of the cellular cholesterol deposits after 12 and 24 h. However, a time-dependent clearance of cholesterol was observed, which thus confirmed previous reports describing a partial functional retention of p.Ile1061Thr NPC1 [19].

### 2.3. Certain NPC1 Variants Display Molecular Damage

**Figure 3:**
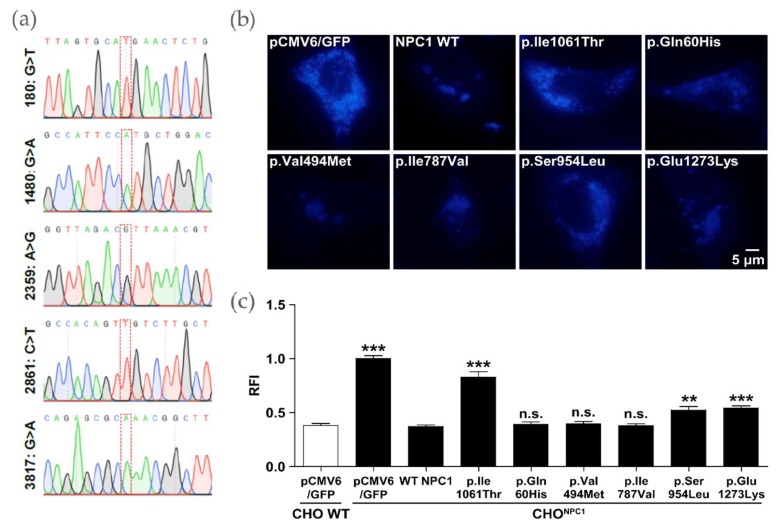
Correction of the cholesterol accumulation phenotype in a complementation test using different *NPC1* variants. (**a**) Sanger sequencing results show the insertion of the desired point mutations into the vector containing NPC1 cDNA. (**b**) Filipin staining. WT NPC1 and different variants were investigated for cholesterol clearance in CHO^Npc1^ cells 12 h post transfection. (**c**) Quantification of the positive cholesterol signal. Values are shown as mean ± SD. Asterisks indicate statistical significance: **p < 0.01, ***p < 0.001.

The NPC1-GFP plasmid constructs were generated using site-directed mutagenesis and subsequently subjected to Sanger sequencing (Eurofins Genomics, Ebersberg, Germany). All constructs showed the desired sequence variant compared to reference sequence NM_000271 and no further unwanted nucleotide exchanges (Figure 3a). The figure shows the area around the respective nucleotide exchange.

**Figure A1:**
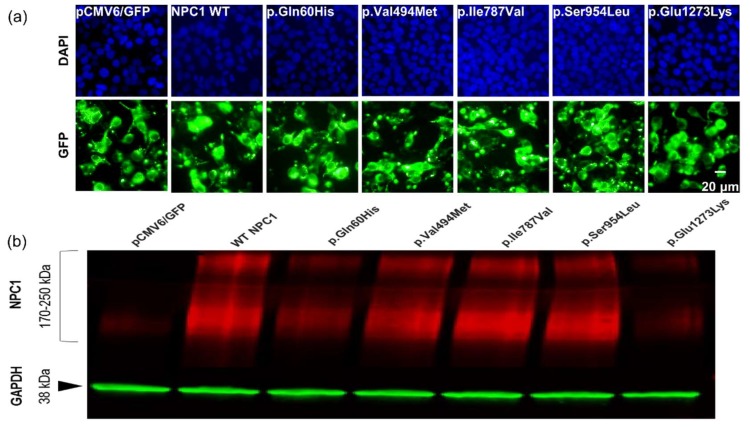
Transfection and expression control experiment in HEK293H cells. (**a**) HEK293H cells were transfected with the NPC1-GFP vector constructs and the respective pCMV6/GFP control vector as indicated. The cells were subjected to fluorescence imaging, generating images for GFP and DAPI 24 h post-transfection. (**b**) Lysates of the transfected HEK293H cells were used for Western blot. NPC1 signal (red) was examined qualitatively optically. GAPDH (green) was used as loading control.

In an initial experiment, it was investigated whether the plasmid constructs led to NPC1 transporter expression. To this end, HEK293H cells were transfected and examined under the fluorescence microscope for NPC1-GFP positive signals (Figure A1a). Western blot analysis further evidenced positive expression of the NPC1-GFP variants as shown by the NPC1 antibody reactive material at >170 kDa 24 h post-transfection (Figure A1b). Control vector pCMV6/GFP transfected cells showed only a low signal which can be assigned to endogenous NPC1 in HEK293H. We further tested the filipin signal in CHO^Npc1^ after 12 h and 24 h post transfection with the respective plasmid constructs. p.Ser954Leu and p.Glu1273Lys showed only partial normalization of the cholesterol elimination from LSOs after 12 h comparable to the p.Ile1061Thr variant (Figure 3b,c). Similar to the situation for p.I1061T after 24 h the Filipin signal is further reduced for p.Ser954Leu, but still shows a significant difference to the WT NPC1 phenotype. The signal for p.Glu1273Lys is completely normalized after 24 h, so that we conclude that these two NPC1 variants maintain transport capacity against cholesterol, but that it is kinetically inferred. The remaining variants p.Gln60His, p.Val494Met, and p.Ile787Val exhibited WT NPC1-like filipin signal after 12 h and 24 h. Therefore, no experimentally justifiable pathological change was detectable for these variants.

### 2.4. 25-Hydroxycholesterol Enhances Cholesterol Elimination from LSOs

**Figure 4:**
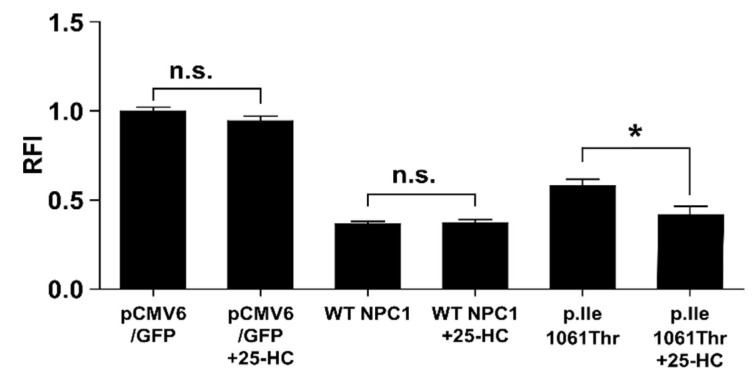
Cholesterol elimination by the p.Ile1061Thr variant can be assisted by 25-hydroxycholesterol. The black bars indicate the filipin signal of untreated or 5 µM 25-hydroxycholesterol treated CHO^NPC1^ cells transfected with either pCMV6 control vector or WT NPC1-GFP vector 24 h post-transfection. Values are shown as mean ± SD. Asterisks indicate statistical significance: *p < 0.05, n.s. = not significant.

We used the established system as a platform to test candidate drugs for the treatment of NP-C. To this end, the efficacy of the pharmacological chaperone 25-hydroxycholesterol (25-HC) [20] was assessed in CHO^Npc1^ cells. 25-HC was unable to reduce cholesterol in cells deficient of NPC1 protein in control vector transfected cells (Figure 4) providing that 25-HC requires a functional NPC1 protein to mediate cholesterol clearance. Transfection of WT NPC1 normalized the filipin signal while 25-HC had no further effects. The partial cholesterol elimination obtained with the p.Ile1061Thr variant was significantly alleviated to WT level by the addition of 25-HC.

## 3. Materials and Methods

### 3.1. Patients

The approval of the ethics committee of the University of Greifswald was obtained for the conduct of the patient-based examinations of the study (file number: BB111/10, date of approval 29 September 2009) [21]. All participants gave written informed consent. A cohort of 149 patients with either non-alcoholic fatty liver disease (*n* = 71), alcoholic-fatty liver disease (AFLD, *n* = 28), or liver cirrhosis (*n* = 50) were genetically screened for gene variants in 330 specific gene loci associated with or potentially predisposing for a liver pathology. Inclusion criteria for the distinct pathologies: LC: Child-Pugh > 4, APRI > 2, characteristic ultrasound findings, no HCV, FL: Characteristic ultrasound findings, APRI < 2, AFLD: Alcohol abuse, BMI < 30, NAFLD: No alcohol abuse, BMI > 35. Likewise, 50 healthy probands were also genetically tested.

### 3.2. Variant Selection

#### 3.2.1. Whole Genome Sequencing and Variant Selection

Roughly 100 ng of genomic DNA prepared from EDTA-blood was used for PCR-free genome sequencing library production (Illumina, San Diego, CA, USA) and resulting libraries have been sequenced using standard parameters on HiSeq X instruments (Illumina): In brief, 2 pmol of sequencing libraries with ~300–400 bp genomic inserts were loaded per flow-cell lane and cBot cluster generation was carried out (Illumina, San Diego, USA). Clustered flow-cells have been sequenced using HiSeq X 2 × 150 bp paired-end chemistry and resulting sequencing data has been converted into fastQ files, mapped against the human reference genome (hg19) using the Illumina pipeline including Isaac alignment, SNV and small indel calling using Strelka, structural variant calling using Manta, variant annotation with our in-house tools, and database resources [22]. The variant list has been quality filtered and mutation burden in genes with a published association with liver disease (OMIM phenotype search with terms like “liver disease”, “liver failure”, “fatty liver disease”) has been assessed by comparing rare protein changing variants in cases and controls. Importantly, the variants selected for the investigation were not present in the AFLD or control cohort, but only found in either the NAFLD or the LC cohort. The variants had low abundance (< 5 entries) in the CentoMD database [22] that contains *NPC1* locus information (CentoMD version 4.0, queried 12/2017). Furthermore, the variants had a low prevalence according to gnomAD (Table 1).

#### 3.2.2. Plasmid Generation

The plasmid vector expressing wild type NPC1-GFP fusion protein (“WT NPC1”), where GFP is fused to the cytosolic C-terminal end of NPC1, was purchased from Origene (Herford, Germany). WT sequence (reference sequence: NM_000271) was confirmed by Sanger sequencing analysis. The mutant constructs were obtained by mutagenesis PCR using the QuikChange II XL Site-Directed Mutagenesis Kit (Agilent Technologies, Waldbronn, Germany). Mutagenesis PCR was performed according to the manufacturer’s manual. Sequence integrity was determined by Sanger sequencing (MWG Eurofins, Munich, Germany) of the obtained plasmid vectors using customized amplification primers to cover the whole coding sequence of the *NPC1* gene. All primer sequences are displayed in Table A1.

#### 3.2.3. Cell Culture

HEK293H and CHO cells were maintained in Dulbecco’s modified Eagle medium containing high glucose supplemented with 1% penicillin-streptomycin (both from Thermo Fisher Scientific, Dreieich, Germany) and 10% fetal bovine serum (GE Healthcare Life Sciences, Munich, Germany). The cells were subcultured by regular splitting 1:15 once they had reached a confluency of 80–90%.

#### 3.2.4. Transfection

HEK293H and CHO cells were seeded in each well of a 24-well plate to achieve 90% cell density on the day of transfection. The cells were allowed to adhere to the bottom of the plates for 24 h. Transfection with the NPC1-GFP fusion gene plasmid constructs was done using Lipofectamin 2000 (Thermo Fisher Scientific, Dreieich, Germany) according to the manufacturer. Six hours after transfection the medium was replaced by fresh DMEM containing all supplements.

#### 3.2.5. Western Blot

The cells were harvested by aspirating the medium and washing with PBS (Biochrom, Berlin, Germany). Then, 200 µl RIPA lysis buffer containing protease inhibitor cocktail (Roche Diagnostics, Penzberg, Germany) were directly added to the wells, and the plate was placed on ice with slight agitation for 20 min. The lysates were subsequently transferred to a 1.5 mL microreaction vial and spun at 14,000× *g* for 15 min at 4 °C in a benchtop centrifuge (Hermle Labortechnik, Wehingen, Germany). The supernatant was recovered and protein concentration was measured using the BCA assay kit (Thermo Fisher Scientific, Dreieich, Germany). 20 µg total protein was mixed with 5× Laemmli buffer and loaded onto a 4–20% Criterion TGX precast midi gel (BioRad, Munich, Germany). The gel was run for 1 h at 200V. Proteins were transferred to a nitrocellulose membrane using the Trans-Blot Turbo transfer system (BioRad). The membrane was shortly rinsed in TBS containing 0.1% Tween (TBS-T) to remove the transfer buffer residue. The membrane was blocked using 5% skim dry milk (Sigma, Steinheim, Germany) and incubated with rabbit monoclonal anti-NPC1 [EPR5209] (abcam, Cambridge, United Kingdom) at a dilution of 1:500 and mouse monoclonal anti-GAPDH [6C5] (abcam) at a dilution of 1:10,000 in TBS-T supplemented with 3% skim dry milk. Each step was followed by extensive washing with TBS-T. Secondary antibodies IRDye^®^ 680LT goat anti-rabbit IgG (H+L) (Li-Cor, Bad Homburg, Germany) and DyLight 800 conjugated goat anti-mouse IgG (H&L) (Thermo Fisher Scientific) in TBS-T containing 3% skim dry milk were applied at a dilution of 1:20,000 and 1:10,000, respectively, for 1 h. After final washing, the membrane was dried and scanned using a Licor Odyssey (Li-Cor). The pictures were analyzed and adjusted for publication using the Odyssey Software version 2.1.

#### 3.2.6. Filipin Staining and Fluorescence Microscopy

CHO cells were seeded on coverslips placed in the wells of a 24-well dish at a concentration of 0.26 × 10^6^/mL. Prior to transfection with Lipofectamin 2000 the cells were allowed to adhere for 24 h. At 12 and 24 h after the transfection, the CHO cells were fixed using 4% PFA in PBS at pH 7.4 for 15 min followed by washing with PBS and incubation with filipin solution (0.5 mg/mL in PBS; Polysciences, Warrington, PA, USA) at room temperature in the dark for 45 min. After extended washing in PBS in a dark chamber, the coverslips were mounted with Mowiol mounting medium (Mowiol, #81831, Sigma) and stored at 4 °C until the analysis. Imaging was carried out using a digital compact microscope (BZ-8000; Keyence, Neu-Isenburg, Germany) with a 40X/NA0.95 objective. Filipin signal was detected with a DAPI-B filter cube (EX, 360/40; DM, 400; and BA, 460/50). Exposure time for filipin: 100 ms; exposure time for GFP: 25 ms. Only NPC1-GFP positive cells were included in cholesterol quantification. In each of three independent experiments, 10 image sections per coverslip were randomly selected and analyzed under constant settings (fluorescence intensity and exposure time). The fluorescent intensity was measured using Image J software (NIH, Bethesda, MD, USA).

#### 3.2.7. Statistical Analysis

Data was analyzed using GraphPad Prism 5 software (GraphPad Software Inc., La Jolla, CA, USA) and represented as mean ± standard deviation (SD). Statistical significance was evaluated using one-way ANOVA and post-hoc Bonferroni test. Statistical significance was defined as *p < 0.05; **p < 0.01; and ***p < 0.001.

## 4. Discussion

NP-C disease is associated with extensive infestation of the liver. The liver is regarded as a key player in cholesterol homeostasis. Therefore, we investigated a cohort of 149 patients with either NAFLD or LC of unexplained etiology for the presence of *NPC1* gene variants. We report the occurrence of mono-allelic *NPC1* gene variants in single individuals. The clinical significance of the variants p.Gln60His (rs145666943), p.Val494Met (rs199812609), p.Ile787Val (rs202046984), and p.Glu1273Lys (rs969680897) is uncertain (Table 1). The variant p.Ser954Leu was previously found in a compound heterozygous patient who demonstrated with late infantile NP-C [23] and is very likely associated with a severe biochemical defect of the NPC1 protein [24]. However, PolyPhen2 [25] and SIFT [26] provide inaccurate predictions as reflected by the contradictory statements shown in Table 1. The different results can be explained by the inclusion of different structure features used by the tools [27]. While these algorithms are generally useful to estimate the evolutionary conservation and thereby the tolerance or intolerance for variant proteins, there is very limited usability for clinical phenotype prediction. Even though the structural elucidation of the NPC1 protein has made progress in recent years, damage predictions by single amino acid substitutions are still vague and impeded by the complex structure of the molecule [28]. Although certain domains (e.g., the cholesterol binding site) are well defined and specific amino acids are of such importance that their loss is equivalent to a loss-of-function mutant [29], GVUS occur frequently. The aim of the study was therefore to develop a highly reproducible model assay based on an isogenic standard cell culture that allows an easy and on demand assessment of the pathogenicity of novel *NPC1* gene variants.

Two of the investigated variants, p.Ser954Leu and p.Glu1273Lys, showed evidence of cholesterol transport impairment, like the common NP-C allele p.Ile1061Thr. P.Glu1273Lys is located at the C-terminal flexible loop in which so far no NP-C disease associated variant has been described and for which no structural data are available, which renders prediction models such as SIFT and PolyPhen2 of little significance. It has been demonstrated that in some cases NP-C patient skin fibroblasts express considerably lower levels of variant NPC1 compared to normal cells [23]. The cells of late infantile patients showed significantly more pronounced effects compared to cells of juvenile or adult onset patients [23]. The p.Ser954Leu displayed no signs of reduced NPC1 protein signal in an initial control experiment in HEK293H cells, while the variants of p.Gln60His and p.Glu1273Lys did (Figure A1b). This may be a first mechanistic evidence of active ER-associated degradation (ERAD) to be responsible for the observed incomplete cholesterol clearance of the p.Glu1273Lys variant. Interestingly, this finding contradicts the general observation that mutations located on the C-terminal part of NPC1 do not interfere with gene expression [23]. The metabolic phenotype of the p.Ser954Leu variant, located within the NPC1 specific cysteine rich domain, does not seem to be due to reduced protein levels. However, these data should not be overestimated as other factors may influence the findings in the assay described here. We have not normalized the NPC1 levels against transfection efficiency. Thus, it can only be speculated that active ERAD is present at this stage. In addition, the GFP fusion of NPC1 can lead to unnatural conformational changes and thus to altered stability [30].

Although the cohort is too small to suggest genetic association, we have identified two kinetically altered variants, p.Ser954Leu and p.Glu1273Lys, in mono-allelic carriers with NAFLD. NP-C disease covers an extremely broad heterogeneous spectrum of symptoms. Especially in the context of aging it is already known that certain subsets of symptoms such as neuropsychiatric and neurodegenerative signs may occur late onset even in *NPC1* mutation carriers [12]. There are indications that carriers of pathogenic variants might also be affected by altered (hepatic) lipid metabolism (reviewed in [31]). Thus, the occurrence of less severe variants or carrier status may predispose to liver damage such as steatosis, fatty liver and cirrhosis. Hence, it should be considered that *NPC1* haploinsuffiency may represent a component of risk for a biochemical deficit patronizing the liver pathology. However, further mutational screenings in cohorts of patients with a liver pathology should be pursued to investigate the potential role of the *NPC1* gene to the observed pathology.

Even though there are few arguments for a genotype-phenotype correlation in NP-C due to the high allelic heterogeneity and the rarity of the disease, metabolic markers (i.e., cholesterol esterification rate) may aid to distinguish genotypes and clinical outcomes in NP-C [18,32]. The system described here provides quantifiable information on the cholesterol transport damage of *NPC1* variants and, thus, helps predict the clinical risk potential and provides extended insights for genotype-phenotype investigations in the future. Moreover, the CHO^NPC1^ cell model is suitable for a small molecule screening to identify drugs with the ability to reduce lysosomal cholesterol [33]. It may also be useful in identifying amenable gene variants for pharmacological chaperone therapy proposed by Ohgane et al. [20].

The NP-C specific therapy with the competitive inhibitor of glucosylceramide synthase miglustat could even be considered for the treatment of these patients. Thus, the assay we present here may be of high clinical relevance to other disorders besides classical Niemann Pick Type C disease.

## 5. Conclusions

There are over 400 *NPC1* variants known to cause NP-C disease. The assay described here offers the possibility of rapid initial characterization of *NPC1* variants to improve diagnostics and prognostics. In addition, the system is suitable for small molecule drug testing.

## Figures and Tables

**Table 1:** Summary of *NPC1* gene variants.

DNA	Protein	Exon	Prediction		Clinical Significance ^1^	Allele Frequency (MAF) ^2^
			PolyPhen2	SIFT		
c.180G>T	p.Gln60His	2	possibly damaging	tolerated	no entry	3.314 × 10^−4^
c.1480G>A	p.Val494Met	9	benign	tolerated	GVUS	1.657 × 10^−4^
c.2359A>G	p.Ile787Val	15	benign	tolerated	no entry	1.450 × 10^−4^
c.2861C>T	p.Ser954Leu	19	possibly damaging	damaging	pathogenic	8.292 × 10^−5^
c.3817G>A	p.Glu1273Lys	25	benign	damaging	no entry	no data

^1^ as referenced in ClinVar [15], ^2^ Allele frequency in exomes of non-Finnish European population according to gnomAD (https://gnomad.broadinstitute.org/).

**Figure A2:**
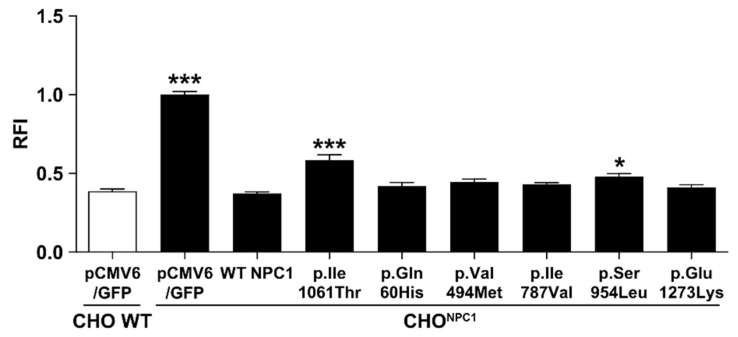
Correction of the cholesterol accumulation phenotype in CHO^NPC1^ cells 24 h post-transfection. Display of the quantification of the positive cholesterol signal. Values are shown as mean ± SD. Asterisks indicate statistical significance: *p < 0.05, ***p < 0.001.

## Appendix A

**Table A1:** Primers for *NPC1* mutagenesis PCR and Sanger sequencing.

Primer Name	Sequence (5′-3′)
NPC1_c.180G>T_fw	TGGATATGACTTAGTGCATGAACTCTGTCCAGGATTC
NPC1_c.180G>T_rev	GAATCCTGGACAGAGTTCATGCACTAAGTCATATCCA
NPC1_ c.1480G>A_fw	CCAGAACAGCCATTCCATGCTGGACCACAAGAA
NPC1_ c.1480G>A_rev	TTCTTGTGGTCCAGCATGGAATGGCTGTTCTGG
NPC1_ c.2359A>G_fw	GTGAGTCTCTTGGGGTTAGACGTTAAACGTCAAGAGAAAAATC
NPC1_ c.2359A>G_rev	GATTTTTCTCTTGACGTTTAACGTCTAACCCCAAGAGACTCAC
NPC1_c.2861C>T_fw	GGGTGAAGCCACAGTTGTCTTGCTGTCGAGT
NPC1_c.2861C>T_rev	ACTCGACAGCAAGACAACTGTGGCTTCACCC
NPC1_ c.3817G>A_fw	ACAAAGGAACAGAGCGCAAACGGCTTCTAAATTTC
NPC1_ c.3817G>A_rev	GAAATTTAGAAGCCGTTTGCGCTCTGTTCCTTTGT
NPC1_ c.3182T>C_fw	GAAGAAAGCCCGACTTACAGCCAGTAATGTCACCG
NPC1_ c.3182T>C_rev	CGGTGACATTACTGGCTGTAAGTCGGGCTTTCTTC
T7_Standard	TAATACGACTCACTATAGG
NPC1_cds_1	GTGAAAGAGTTACAATACTACG
NPC1_cds_2	CCCATCGATAGCAATATAGC
NPC1_cds_3	CACCGTATAACACGAACTGC
NPC1_cds_4	GCTGAAGATGGAACAAGCGT
NPC1_cds_5	AGTGGTTGACCCTGCCTGCGTT
NPC1_cds_6	TTGGAGTTATGTGGCTCTGG
